# Formation of luminescent defects in 4H-SiC upon ion irradiation and ns laser annealing

**DOI:** 10.1038/s41598-025-23259-6

**Published:** 2025-11-12

**Authors:** Gabriele Zanelli, Igor Fontan, Greta Andrini, Emilio Corte, Elena Nieto Hernández, Veronica Varzi, Nour-Hanne Amine, Sofia Sturari, Elisa Redolfi, Valentino Rigato, Matteo Campostrini, Paolo Traina, Federico Picollo, Marco Genovese, Sviatoslav Ditalia Tchernij, Jacopo Forneris

**Affiliations:** 1 Physics Department, University of Torino, Torino, 10125 Italy; 2 Italian National Institute of Metrological Research (INRiM), Torino, 10135 Italy; 3 Italian National Institute of Nuclear Physics (INFN) - Sez. Torino, Torino, 10125 Italy; 4 Italian National Institute of Nuclear Physics (INFN) - Laboratori Nazionali di Legnaro, Legnaro, 35020 Italy

**Keywords:** Single-photon sources, Ion implantation, Silicon carbide, Silicon vacancy, Photoluminescence, Formation yield, Laser annealing, Color centers, Optical materials and structures, Lithography, Design, synthesis and processing

## Abstract

We present a novel approach for the optical activation of the negatively-charged silicon vacancy ( V_Si_^−^) center in ion irradiated silicon carbide (SiC) via ns-pulsed laser annealing in the 234–2180 mJ cm^− 2^ energy density range. The laser annealing process is investigated under 355 nm and 532 nm wavelengths at pulse energy densities below the melting threshold and validated by means of Raman spectroscopy and photoluminescence mapping. The combined effect of ns pulsed laser annealing and subsequent thermal treatment is also assessed. The results offer a promising resource for the development of integrated photonic SiC devices and could be extended to a potentially wide range of applications involving other classes of solid-state quantum emitters.

## Introduction

In recent years solid-state optically active defects, also referred to as *color centers*, emerged as a promising platform for Single Photon Sources (SPS) development, offering system- and platform-dependent emission from the UV to the IR range of the electromagnetic spectrum, with enticing applications in quantum-enhanced cryptography, computing and sensing^1–3^. Besides defect-specific opto-physical properties, such as emission intensity, quantum efficiency and optically-addressable spin states already consolidated in diamond platform (NV, GeV, SnV)^4–6^, their general availability in wide band-gap semiconductors^7–11^ implies the possibility of room-temperature operation, combined with the availability of a solid-state host matrix guaranteeing a robust and easily-manufacturable substrate. In this regards, silicon carbide (SiC) has gained increasing interest due to extreme physical properties, such as excellent optical, thermal and electrical properties^12,13^, combined with a mature synthesis and manufacturing technology^14^ leading to the realization of SiC-based photonic and integrated devices^12^.

Extrinsic color centers in SiC, primarily Cr^4+^ and V^4+^ and based defects, have been recently investigated due to their optical emission at different telecom wavelengths^15,16^. However, these color centers currently require cryogenic temperature operativity and dedicated ion implantation fabrication processes. Conversely, intrinsic color centers in SiC are easier to fabricate by means of the controlled introduction of lattice disorder by ion^17^, electron^18^ or photon^19^ irradiation. Among them, the most studied impurity complex is the negatively charged silicon vacancy (V_Si_^−^), offering spin-dependent fluorescence and addressability^20^, short optical lifetime (~ 6 ns) and NIR photoluminescence (PL) emission, together with room-temperature operation^8^. Coherent control of the spin state of the defect in an isotopically purified sample shows long inhomogeneous spin dephasing times (T_2_^*^=30 µs) which is one order of magnitude lower to the values reported for NV centers in ultrapure diamond (T_2_^*^=470 µs)^21^. The V_Si_^−^ center is currently employed as a temperature and magnetic field sensor^22^ reaching sensitivities down to 4 nT/Hz^− 1/2^^23,24^. The fabrication of the V_Si_^−^ center is performed primarily by the creation of lattice vacancies^8^. Subsequently, thermal annealing at 400–600 °C in controlled atmosphere is required to restore undesired defective complexes and activate the V_Si_^−^ emission^25^. However, this annealing method presents a low activation yield (~ 4%)^26^ and lacks spatial control on the sample region exposed to the process. Other techniques, such as femtosecond-laser writing, have succeeded to demonstrate the scalable fabrication of single V_Si_^−^ centers^27^. However, this method induces lattice damage and high local temperatures that can be detrimental to the properties of the fabricated color centers or of the additional surrounding features hosted by the material substrate, such as electrical dopants or metallic electrodes.

In this work, we propose an approach for the optical activation of the V_Si_^−^ center in ion irradiated SiC, with a potentially wide range of application to other classes of solid-state quantum emitters. This method relies on the selective annealing of µm-sized regions of the material upon ns-pulsed laser heating. While pulsed laser annealing of SiC is a widely employed tool for the annealing of radiation damaged SiC^28^ and the electrical activation of shallow dopants^29,30^, this approach has not been explored so far for the activation of photoluminescent defects. The effectiveness of this method to produce V_Si_^−^ centers upon localized, non-destructive annealing is here demonstrated by means of Raman spectroscopy and PL mapping. The effects of different lasing wavelengths and processing parameters in the formation of the afore-mentioned color centers is also investigated.

## Experimental methods

The sample analyzed in this work was a 350 μm thick High Purity Semi-Insulating 4H-SiC wafer purchased from Wolfspeed (HPSI, Resistivity ≥ 1 · 10^6^ Ω cm ). Two fragments of the sample were irradiated with a broad homogeneous beam of 1 MeV He^2+^ ions at a F = 1·10^15^ cm^− 2^ and F = 5·10^15^ cm^− 2^ fluences, (Sample #1 and Sample #2, respectively). Care was taken to mask a portion of the sample with a ~ 20 μm thick Al topping layer to prevent beam exposure, thus leaving a pristine control region of the sample unirradiated for the subsequent characterization.

The pulsed laser annealing was performed on the irradiated areas of both samples using a Nd:YAG source (355 nm and 532 nm wavelengths) operating in “Q-switching” mode, with a maximum achievable output energy of 0.6 mJ within a nominal pulse duration of 4 ns and a repetition rate of 50 Hz. The beam was delivered to the laser output through a motorized collimating aperture enabling to define a 25 × 25 µm^2^ and 12 × 12 µm^2^ lased regions on Sample #1 and Sample #2, respectively. The laser was delivered via coupling with an optical microscope equipped with 50X and a 20X magnification objectives for the 355 nm and 532 nm sources, respectively, resulting in a maximum achievable energy density of 24000 mJ cm^− 2^ and 3840 mJ cm^− 2^. A motorized variable thickness filter was employed to control the pulse energy density in the 0-100% range. The two lasing wavelengths enabled to explore photon absorption processes both below (2.33 eV) and above (3.49 eV) the energy gap threshold of 4H-SiC (3.2 eV)^31^.

Additionally, thermal annealing in N_2_ inert atmosphere^8^ was carried out at different temperatures (300 °C, 450 °C, 600 °C, 750 °C, 900 °C) for 30 min on Sample #1 following the ns pulsed laser annealing. The goal of this last processing step was to compare the effects of the two processes separately as well as to investigate their combination. The temperature range was chosen to prevent surface roughening and sublimation occurring above 1000 °C^32^. Different classes of defects present different optimal values of annealing temperature to achieve the best activation yield. For the V_Si_^−^ color center the optimal value is 600˚C, for CAV^+^ is 1000˚C, and for VV^0^ the optimal temperature is 900˚C^33^. The processed samples were characterized via Raman and PL spectroscopy after ns-pulsed laser annealing and after each thermal annealing step using a *Horiba Jobin Yvon HR800* confocal micro-spectrometer (633 nm excitation laser wavelength, 20 mW optical power) with a 0.1 nm resolution. The spatial distribution of the PL intensity was assessed by means of a custom single-photon-sensitive confocal microscope^34^ equipped with a 660 nm CW laser source (300 µW optical power) and a 100x dry objective (0.9 N.A.).

## Results

### Laser annealing effects

**Fig. 1:**
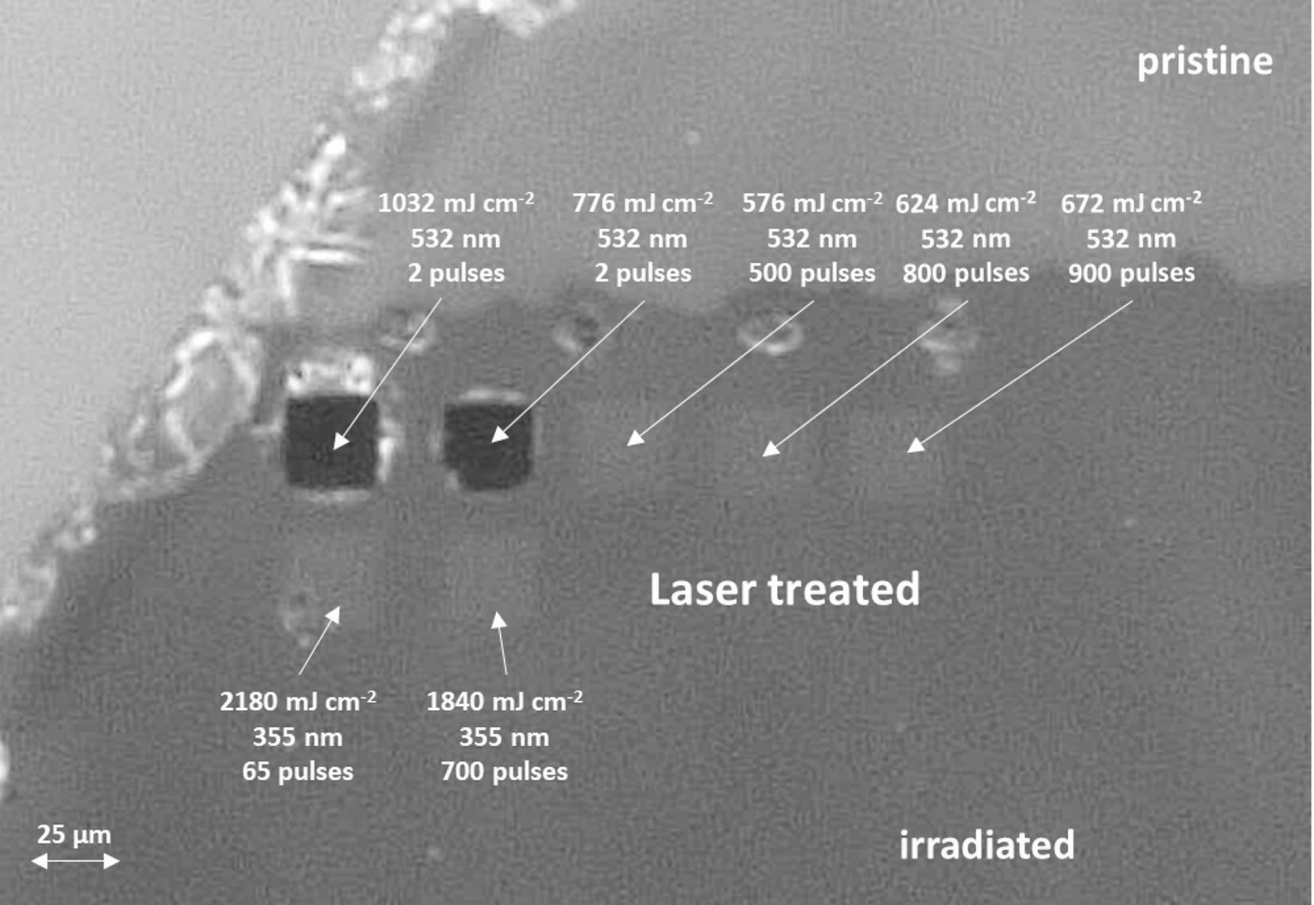
Widefield micrograph of Sample #1 showing: untreated region of the sample (pristine), the 1 MeV He^2+^ irradiated region at a F = 1·10^15^ cm^− 2^ (irradiated), the portions of the irradiated regions that underwent pulsed-laser processing (Laser treated) with relevant processing parameters indicated for each region.

A preliminary inspection of Sample #1 following ion irradiation and laser processing was performed by optical microscopy (Fig.1). The portion of the sample exposed to MeV ion irradiation (lower part of the micrograph) appears more opaque with respect to the pristine material (upper part) as a consequence of the formation of absorption centers. Several squares (with corresponding processing parameters indicated in Fig. 1) are visible in the irradiated region, corresponding to the spots where the ns laser pulses were delivered. The two regions irradiated with the largest energy density (processed at 776 mJ cm^− 2^ and 1032 mJ cm^− 2^ at 532 nm wavelength, respectively) were clearly structurally modified after just 2 laser pulses, showing a major reduction in their optical transmission and apparent surface damage surrounding the irradiated region. Additional laser processing steps were performed with lower energy densities (576 mJ cm^− 2^, 624 mJ cm^− 2^, 672 mJ cm^− 2^) using 532 nm wavelength, however, structural damage was not observed under these conditions. This observation indicates that the melting threshold for the 532 nm wavelength is placed between 672 mJ cm^− 2^ and 776 mJ cm^− 2^. This result is compatible with previous reports on the detrimental effects, such as surface melting and polycrystalline lattice recovery, occurring at 600 mJ cm^− 2^ laser energy densities using a 532 nm wavelength after N^2+^ ion implantation^35^. Noticeably, only minor lattice damage was observed under 355 nm lasing, even though larger power densities were selected (2180 mJ cm^− 2^ and 1840 mJ cm^− 2^). This observation could be explained considering that the 355 nm radiation is absorbed from the entire bulk SiC rather than selectively from the radiation-induced absorption centers which are responsible for the 532 nm absorption.

**Fig. 2:**
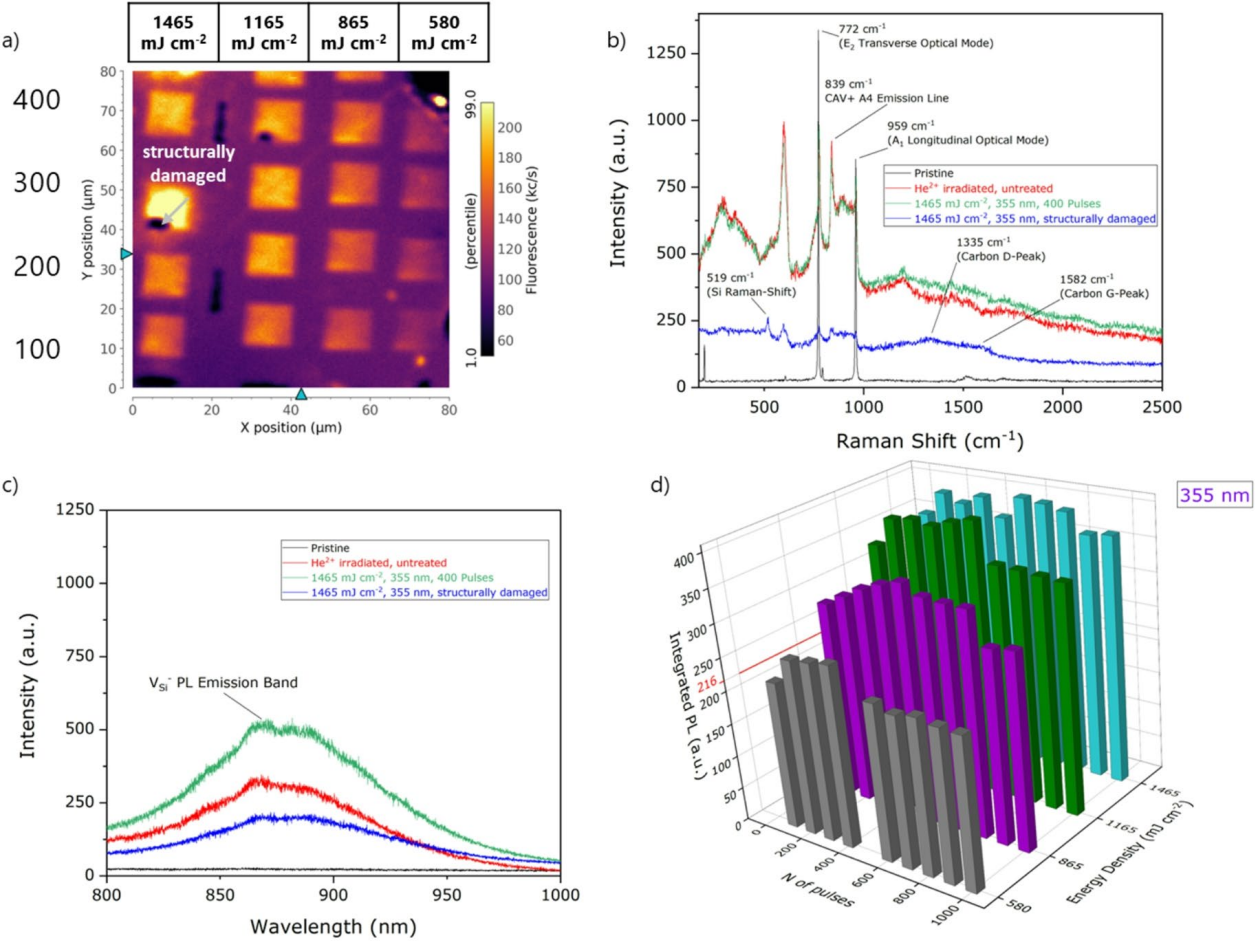
(**a**) PL confocal maps of the 355 nm laser processed regions of Sample #2 with highlighted structurally-damaged region. (**b**) Raman and (**c**) PL spectra of the 355 nm laser processed regions of Sample #2. (**d**) Integrated PL emission of the V_Si_^−^ color center (800–900 nm) as a function of the laser processing power density and number of pulses.

**Fig. 3:**
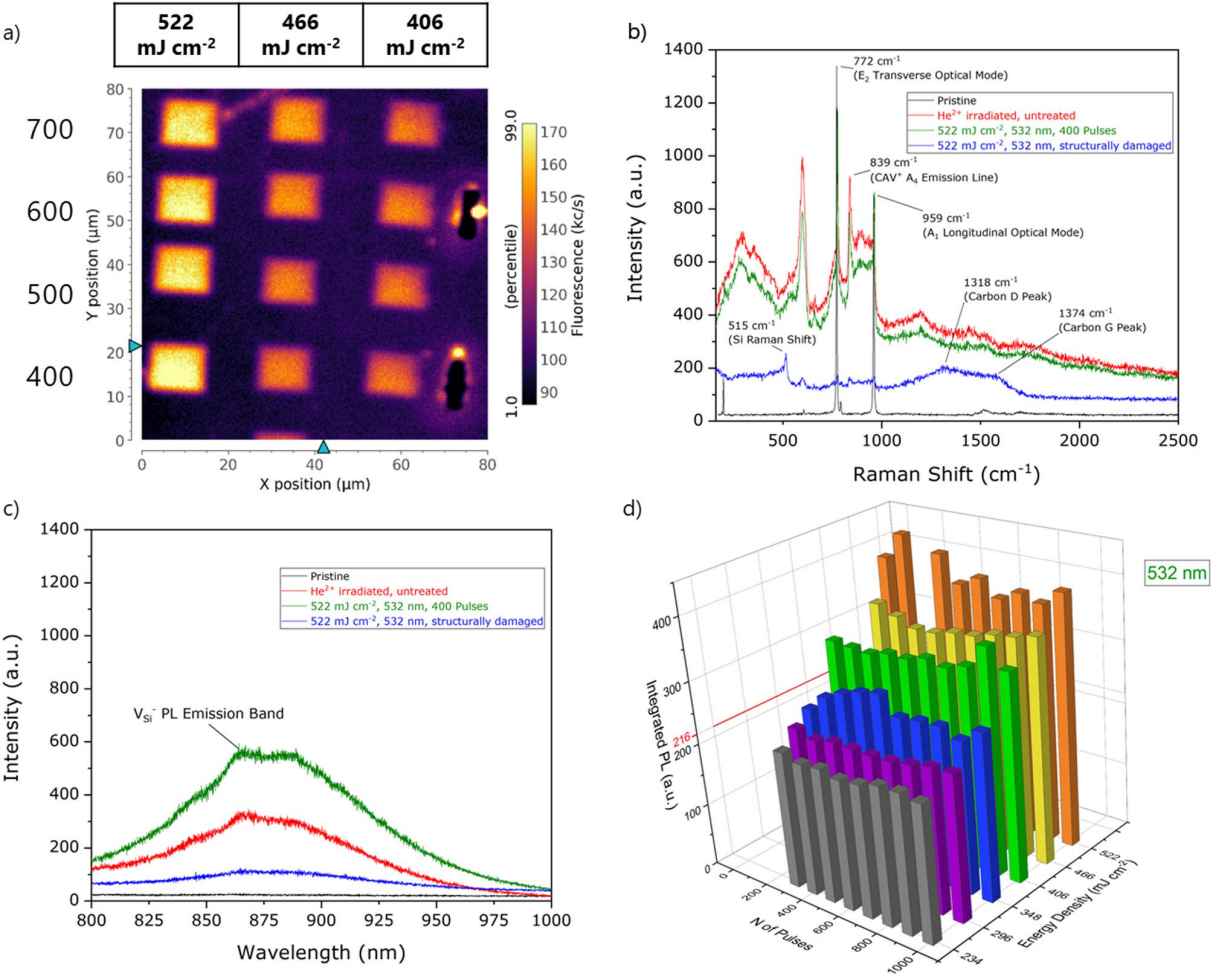
(**a**) PL confocal maps of the 532 nm laser processed regions of Sample #2. (**b**) Raman and (**c**) PL spectra of the 532 nm laser processed regions of Sample #2. (**d**) Integrated PL emission of the V_Si_^−^ color center (800–900 nm) as a function of the laser processing power density and number of pulses.

A more systematic investigation on the laser processing parameters, namely wavelength, energy density and number of pulses, was performed on Sample #2 for pulse energy density below 600 mJ cm^− 2^ for the 532 nm wavelength and 1.5 J cm^− 2^ for the 355 nm wavelength, which have been shown, on the basis of numerical simulation, to define the threshold values for surface modification phenomena such as melting, ablation and recrystallisation^35,36^. Different regions were treated using linearly spaced values of energy densities in the (580–1465) mJ·cm^− 2^ and (234–522) mJ·cm^− 2^ range for the 355 nm and 532 nm lasing wavelengths, respectively. For each value of energy density 10 squares were processed with an increasing number of delivered lasing pulses (100–1000). The results of the laser processing of Sample #2 are reported in Figs. 2 and 3. A confocal microscopy assessment of the emission intensity from optically active defects was performed by PL mapping. Exemplary PL maps are presented in Figs. 2a and 3a encoding, for the 355 nm and 532 nm processing wavelength, the photon emission intensity integrated in the 700 –1000 nm spectral range as a function of the spatial position of the 660 nm laser probe. The maps clearly exhibit a PL intensity increase in the ns laser annealed regions with respect to the surrounding, ion irradiated region. This effect is qualitatively similar for both 355 nm and 532 nm processing wavelengths. Moreover, heat affected zones (HAZ), i.e. modification of the crystalline structure by laser processing without surface melting^37,38^, have not been observed working below the melting threshold, indicating that the localized heat induced by the ns-laser pulse is enhancing the mobility of vacancies without altering the local crystalline structure. Conversely, occasional structural damage is observed at the edge of some lased regions at energy densities close to the melting threshold, as shown in Fig. 2a. This effect is attributed to local fluctuations in the lasing energy density or in the structural damage occurred during the ion irradiation process. The PL intensity acquired at the center of each lased region is reported in Figs. 2d and 3d as a function of the pulse energy density and of the number of pulses delivered, showing a clear increasing trend with energy density, with a > 2-fold enhancement for both processing wavelengths in the considered parameter ranges. Conversely, no apparent dependence of the PL intensity on the number of lasing pulses could be observed. This latter result is consistent with the short duration of the lasing pulses with respect to their repetition rate. The possible occurrence of temperature transients was recently evaluated to be < 1 µs in silicon under similar lasing conditions^39^; the significantly higher thermal conductivity of SiC ensures that, in this case, the time scale for this effect is at most of the same order of magnitude.

To attribute the origin of the PL intensity increase to specific photon emitting defects, Raman and PL spectra were acquired from the laser annealed regions. The Raman spectra are shown in Figs. 2b and 3b for the two laser processing wavelengths in the case of pristine, irradiated and laser annealed SiC. In the latter case, the results for the above-considered highest energy densities below the melting threshold are reported. The Raman spectrum acquired from the structurally damaged region under 355 nm processing is also shown in Fig. 2b.

All the collected Raman spectra show the characteristic features of 4H-SiC at 203 cm^− 1^, 776 cm^− 1^, 962 cm^− 1^^40^(Figs. 2b and 3b). Additional peaks could be observed from the structurally damaged region, corresponding to the formation of pure Si (520 cm^− 1^) and pure C phases (1341 cm^− 1^ and 1585 cm^− 1^). These features further confirm the structural modification of SiC occurring under the processing laser pulses (1165 mJ cm^− 2^ and 1465 mJ cm^− 2^)densities. This value is compatible with the reported melting threshold for UV ns-pulsed laser of 1 J cm^− 2^^41,42^. Furthermore, the A4 PL emission of the CAV^+^ color center at 668.5 nm (corresponding to a 839 cm^− 1^ Raman shift in Figs. 2b and 3b)^5^, is observed following both ion irradiation and all the laser processing conditions below the melting threshold. The intensity of such emission line does not depend on the processing condition, suggesting that the PL increase upon laser annealing observed in Fig. 3a cannot be ascribed to this spectral feature.

The increase in the emission intensity upon PL mapping was conversely found to correlate with the the V_Si_^−^ emission (Figs. 2c and 3c). The V_Si_^−^ signal, which is absent in the pristine region of the sample, appears upon ion irradiation and increases in intensity upon laser annealing. As a further confirmation of severe structural damage, the V_Si_^−^ peak intensity is dramatically reduced in the regions processed at 355 nm energy density above the melting threshold. To clarify this point, Figs. 2c and 3c also include spectra acquired from structurally damaged points of the sample such as that highlighted in Fig. 2a.

### Laser-induced and thermal annealing

The pulsed laser annealing process was combined with a subsequent conventional thermal treatment in order to assess the joint effect of the two methods in the formation of the V_Si_^−^ center.

**Fig. 4:**
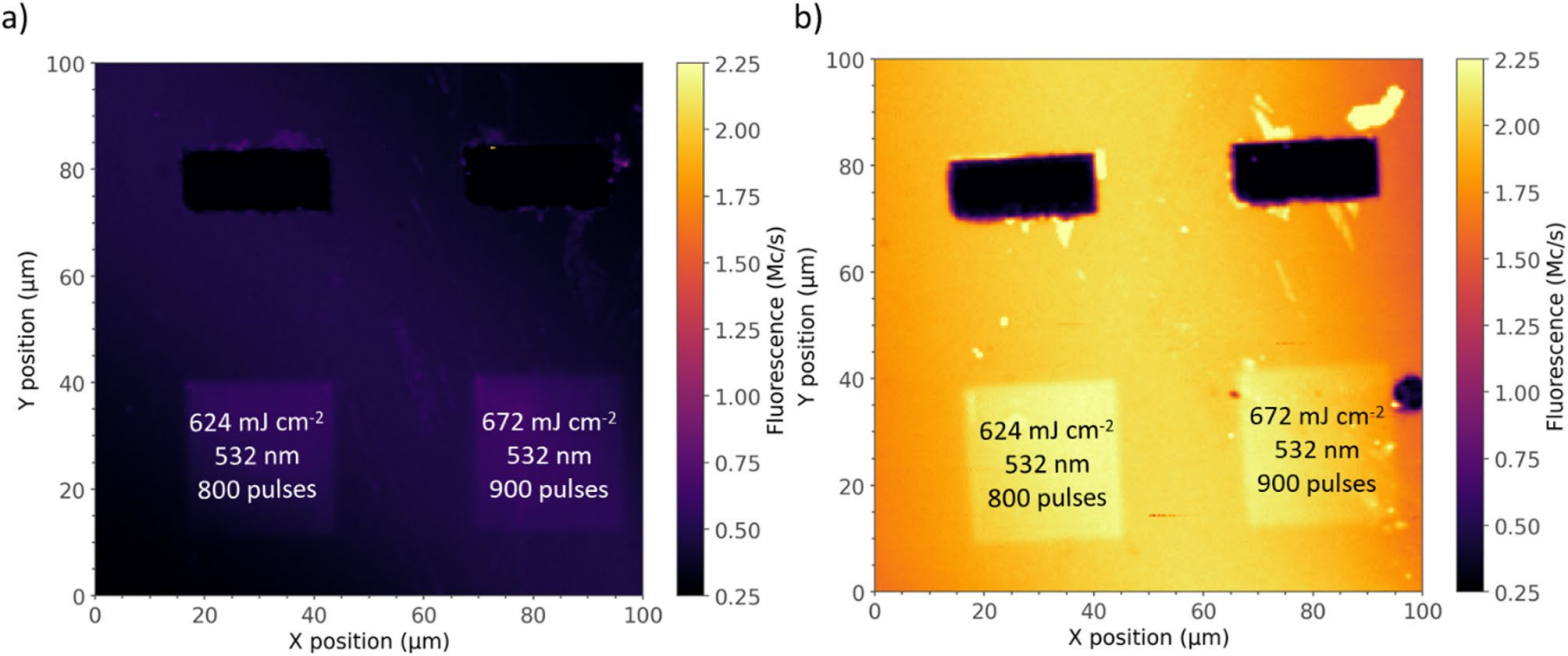
(**a**) Confocal PL map of two laser processed regions; (**b**) Confocal PL map of the same regions following a further 450 °C thermal annealing in N2 atmosphwere for 30 min.

Figure 4a reports a PL map of two exemplary regions processed by 532 nm laser annealing at energy densities below the melting threshold. The relevant processing parameters adopted for the 532 nm lasing are reported in the labels. Figure 4b shows the regions following a subsequent thermal annealing, performed at 450 °C in N2 atmosphere for 30 min. For consistency, the two PL maps are shown with the same color scale encoding the PL emission intensity. Consistently with the previous discussion, the sole laser processing (Fig. 4a) evidenced an apparent increase in the PL signal related to the VSi^−^ center (up to ~ 800 kcps PL intensity) with respect to the surrounding irradiated material (~ 600 kcps), separated by sharp and well defined edges. Following a 450 °C thermal treatment, the PL signal increases in both the laser processed regions (up to ~ 2.25 Mcps PL intensity) and in the solely irradiated material (~ 1.6 Mcps). This result indicates that following thermal treatment a higher emission intensity is still observable in the laser processed regions, thus suggesting the potential enhancement in the formation of the VSi^−^ center upon the combination of both post-implantation steps.

**Fig. 5:**
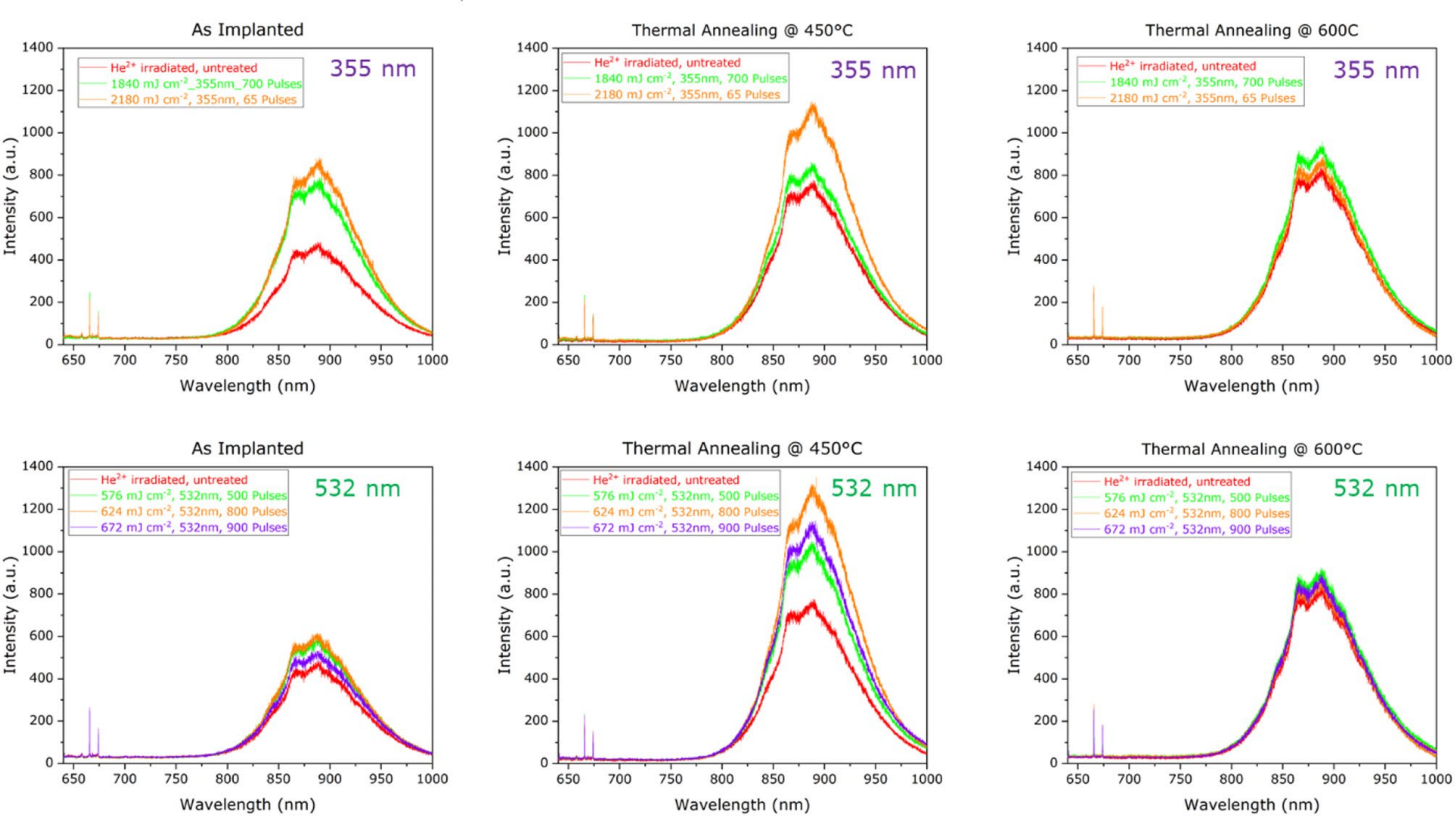
V_Si_^−^ PL spectra collected from laser processed regions with (**a**) 532 nm and (**b**) 355 nm laser wavelengths of sample #3, which have subsequently undergone no thermal treatment, 450 °C thermal annealing, 600 °C thermal annealing.

**Table 1:** V_Si_^−^ PL intensity values for different thermal processes (no thermal processing, 450 °C thermal annealing, 600 °C thermal annealing) for the laser untreated region and the two laser processed regions providing the largest PL signal for the two laser annealing (LA) wavelengths (532 nm, 624 mJ cm^− 2^, 800 pulses and 355 nm, 2180 mJ cm^− 2^, 600 pulses).

Integrated PL intensity			
	No thermal treatment	450° C, 30 min	600 °C, 30 min
No LA	450	750	850
LA 532 nm, 624 mJ cm^− 2^, 800 pulses	600	1300	850
LA 355 nm, 2180 mJ cm^− 2^, 600 pulses	850	1150	850

**Fig. 6:**
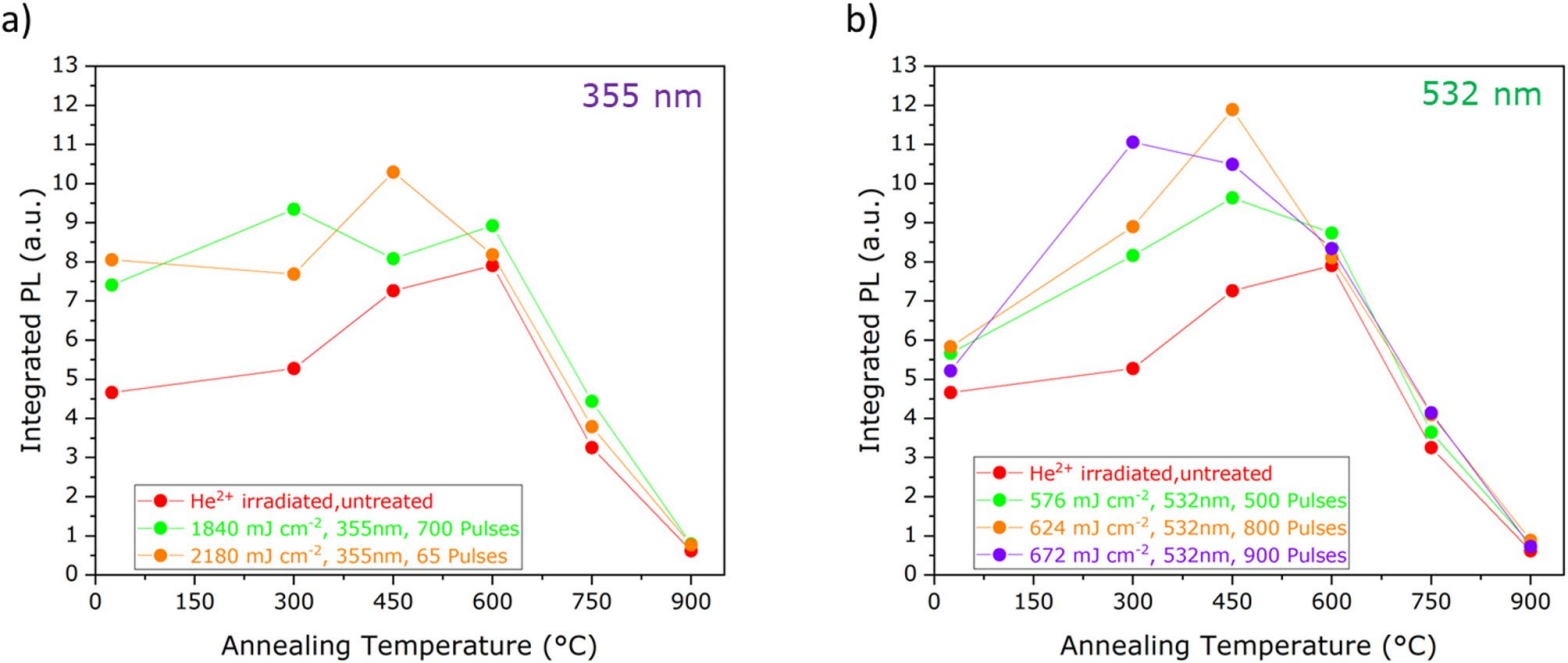
Integrated PL (from 800–1000 nm) of the V_Si_^−^ color center as a function of the different thermal annealing temperatures for (**a**) 355 nm laser processed and (**b**) 532 nm laser processed regions.

This effect is further investigated in Fig. 5, where PL spectra acquired from regions lased both under 532 nm and 355 nm are shown following pulsed laser annealing and subsequent different thermal annealing at 450 °C and 600 °C temperatures. The red line refers to the PL spectra acquired from the control irradiated region that did not undergo pulsed laser annealing, to be compared with those obtained under different lasing energy densities. Before thermal annealing, both the regions processed with 355 nm and 532 nm laser present a larger PL intensity with respect to the irradiated control region. After the thermal annealing step at 450 °C the laser processed regions present a significant increase in the PL intensity, while that acquired from the control irradiated region remains almost unaltered. Following a further 600 °C thermal annealing, the PL signal of the control region increases as well, and all the PL spectra converge to the same PL intensity independently of the laser annealing process previously performed. However, it is worth noting that such intensity is lower with respect to what was measured from the laser processed regions following 450 °C thermal treatment. To further clarify this point, the main results from Fig. 5 are listed in Table 1, while the integrated PL signal over the whole emission spectrum is plotted in Fig. 6 as a function of the different thermal processing treatments in the 300–900 °C range.

The control curve (red, referred to thermal processing only in an ion irradiated region) shows an increasing behavior in the PL intensity up to a 600 °C temperature, followed by a decrease at higher values. This result is in agreement with the results reported in previous literature and indicates that at higher annealing temperatures the vacancy mobility leads to the formation of CAV^+^ and VV^0^ color centers at the expense of the V_Si_^−^ center^25^. Conversely, the 355 nm wavelength laser processing results in a maximum V_Si_^−^ PL emission intensity following the 450 °C thermal annealing step. This result indicates that the temperature needed to enhance the formation of the V_Si_^−^ defects is decreased when the thermal processing of the sample follows a pulsed laser annealing treatment. This effect is even more noticeable in the case of 532 nm laser processing. In this case, the temperature at which the maximum V_Si_^−^ emission intensity occurs clearly depends on the laser pulse energy density. In this case, the lowest temperature value is obtained for the largest processing density. The optimal temperature is found to be lowered from 600 °C (control curve) to 450 °C and 300 °C under 576 mJ cm^− 2^ and 672 mJ cm^− 2^ pulses, respectively. This finding indicates that the laser pre-processing of irradiated SiC enables to obtain an efficient activation of the V_Si_^−^ center at lower temperatures, and thus that the processing history of the material plays a role in the structural reorganization of the lattice upon thermal treatments.

## Conclusions

In this work we presented a novel approach for the optical activation of color centers in SiC via ns-pulsed laser processing using 2 different wavelengths (355 –532 nm) below the melting threshold. Differently from fs laser writing^19,27^, the proposed technique does not introduce additional structural damage to the SiC lattice, instead it delivers localized heat to the material (355 nm) or to the radiation-induced absorption centers (532 nm) to promote the reorganization of the crystal. This method, applied so far only to dopants activation, evidenced an increase in the PL emission from a MeV ion irradiated, high-purity 4H-SiC crystal. Such an increase was demonstrated to originate from an increase in the density of V_Si_^−^ centers. The 355 nm lasing wavelength proved to be more effective than the 532 nm processing, arguably due to the fact that the photon energy (3.49 eV) is larger than the optical bandgap of the material and to the more limited structural effects on the material upon the delivery of large energy densities.

A dependence of the PL emission intensity as a function of the lasing pulses delivered to the sample was not observed, indicating that the activation is achievable already by a few pulses. A further investigation of the technique at larger pulse repetition rates (up to MHz) could clarify whether a more significant dependence of the lasing process outcome on the number of pulses could arise due to temperature build up in the material. A dedicated study on 4H-SiC implanted at low ion fluences will also be crucial to assess the effectiveness of the technique to activate individual V_Si_^−^ centers for quantum information processing and quantum sensing purposes and its role in the coherence time of the electron spin of the defect^43^. Finally, the laser annealing technique was combined with a subsequent thermal annealing process, displaying a lasing pulse energy density-dependent reduction in the optimal annealing temperature (from 600 °C down to 300 °C on 532 nm lased SiC) required to maximize the formation of the V_Si_^−^ center. The possibility to obtain stable, optically active color centers at temperatures significantly lower than those achievable exclusively by means of thermal processing could prove to be an enabling technique for the fabrication of opto-electronic devices conceived for quantum sensing and quantum information processing, in which an electrical stimulation or readout of quantum emitters is achieved by means of integrated microelectrodes^44–46^. The decrease in the required processing temperatures would in fact hinder the formation of new crystallographic phases onto metallic pads deposited on the device that reduce the performance of the electrical contacts^47,48^ and enable the selective, localized activation of the emitters at specific regions of interest registered to the geometry of the driving electrodes.

## Data Availability

The datasets used and/or analysed during the current study available from the corresponding author on reasonable request.

## References

[1] Beveratos, A. et al. Single photon quantum cryptography. *Phys. Rev. Lett.* **89**, 187901 (2002). 12398636 10.1103/PhysRevLett.89.187901

[2] Pezzagna, S. et al. Quantum computer based on color centers in diamond. *Appl. Phys. Rev.* **8**, (2021).

[3] Glenn, D. R. et al. High-resolution magnetic resonance spectroscopy using a solid-state spin sensor. *Nature* **555**, 351–354 (2018). 29542693 10.1038/nature25781

[4] Doherty, M. W. et al. The nitrogen-vacancy colour centre in diamond. *Phys. Rep.* **528**, 1–45 (2013).

[5] Iwasaki, T. et al. Germanium-Vacancy single color centers in diamond. *Sci. Rep.* **5**, 12882 (2015). 26250337 10.1038/srep12882 PMC4528202

[6] Tchernij, S. D. et al. Single-Photon-Emitting optical centers in diamond fabricated upon Sn implantation. *ACS Photonics*. **4**, 2580–2586 (2017).

[7] Aharonovich, I. et al. Solid-state single-photon emitters. *Nat. Photonics*. **10**, 631–641 (2016).

[8] Castelletto, S. et al. Silicon carbide color centers for quantum applications. *J. Physics: Photonics*. **2**, 022001 (2020).

[9] Abdi, M. et al. Color centers in hexagonal Boron nitride monolayers: A group theory and Ab initio analysis. *ACS Photonics*. **5**, 1967–1976 (2018).

[10] Shevitski, B. et al. Blue-light-emitting color centers in high-quality hexagonal Boron nitride. *Phys. Rev. B*. **100**, 155419 (2019).

[11] Berhane, A. M. et al. Photophysics of GaN single-photon emitters in the visible spectral range. *Phys. Rev. B*. **97**, 165202 (2018).

[12] Xing, P. et al. CMOS-Compatible PECVD silicon carbide platform for linear and nonlinear optics. *ACS Photonics*. **6**, 1162–1167 (2019).

[13] Choyke, W. J. et al. Physical properties of SiC. *MRS Bull.* **22**, 25–29 (1997).

[14] Nakamura, D. et al. Ultrahigh-quality silicon carbide single crystals. *Nature* **430**, 1009–1012 (2004). 15329716 10.1038/nature02810

[15] Diler, B. et al. Coherent control and high-fidelity readout of chromium ions in commercial silicon carbide. *Npj Quantum Inform.* **6**, 11 (2020).

[16] Wolfowicz, G. et al. Vanadium spin qubits as Telecom quantum emitters in silicon carbide. *Sci. Adv.* **6**, (2020). 10.1126/sciadv.aaz1192 PMC7195180 32426475

[17] Calusine, G. et al. Silicon carbide photonic crystal cavities with integrated color centers. *Appl. Phys. Lett.* **105**, (2014).

[18] Widmann, M. et al. Coherent control of single spins in silicon carbide at room temperature. *Nat. Mater.* **14**, 164–168 (2015). 25437256 10.1038/nmat4145

[19] Chen, Y. C. et al. Laser writing of scalable single color centers in silicon carbide. *Nano Lett.* **19**, 2377–2383 (2019). 30882227 10.1021/acs.nanolett.8b05070

[20] Nagy, R. et al. High-fidelity spin and optical control of single silicon-vacancy centres in silicon carbide. *Nat. Commun.* **10**, (2019). 10.1038/s41467-019-09873-9 PMC6486615 31028260

[21] Maurer, P. C. et al. Room-Temperature quantum bit memory exceeding one second. *Science* **336**, 1283–1286 (2012). 22679092 10.1126/science.1220513

[22] Kraus, H. et al. Magnetic field and temperature sensing with atomic-scale spin defects in silicon carbide. *Sci. Rep.* **4**, 5303 (2015). 10.1038/srep05303 PMC4081891 24993103

[23] Abraham, J. B. S. et al. Nanotesla magnetometry with the silicon vacancy in silicon carbide. *Phys. Rev. Appl.* **15**, 064022 (2021).

[24] Lekavicius, I. et al. Magnetometry based on Silicon-Vacancy centers in isotopically purified 4H-SiC. *Phys. Rev. Appl.* **19**, 044086 (2023).

[25] Rühl, M. et al. Controlled generation of intrinsic near-infrared color centers in 4H-SiC via proton irradiation and annealing. *Appl. Phys. Lett.* **113**, 122102 (2018).

[26] Wang, J. et al. Scalable fabrication of single silicon vacancy defect arrays in silicon carbide using focused ion beam. *ACS Photonics*. **4**, 1054–1059 (2017).

[27] Castelletto, S. et al. Photoluminescence in hexagonal silicon carbide by direct femtosecond laser writing. *Opt. Lett.* **43**, 6077 (2018). 30548008 10.1364/OL.43.006077

[28] Pecholt, B. et al. Review of laser microscale processing of silicon carbide. *J. Laser Appl.* **23**, 012008 (2011).

[29] Boutopoulos, C. et al. Laser annealing of al implanted silicon carbide: structural and optical characterization. *Appl. Surf. Sci.* **253**, 7912–7916 (2007).

[30] Wu, J. et al. Pulsed laser annealing of Phosphorous-Implanted 4H-SiC: electrical and structural characteristics. *J. Electron. Mater.* **51**, 172–178 (2022).

[31] Kimoto, T. et al. *Fundamentals of Silicon Carbide Technology* (Wiley, 2014).

[32] Canino, M. et al. 4H-SiC surface morphology after al ion implantation and annealing with C‐cap. *J. Microsc.* **280**, 229–240 (2020). 32495384 10.1111/jmi.12933

[33] Calabretta, C. et al. Laser annealing of P and al implanted 4H-SiC epitaxial layers. *Materials* **12**, 3362 (2019). 31618862 10.3390/ma12203362 PMC6829506

[34] Corte, E. et al. Magnesium-Vacancy optical centers in diamond. *ACS Photonics*. **10**, 101–110 (2023). 36691430 10.1021/acsphotonics.2c01130 PMC9855000

[35] Brink, D. J. et al. Quantitative analysis of the lattice reconstruction of ion-implanted SiC after visible light laser irradiation. *J. Appl. Phys.* **105**, (2009).

[36] An, H., Wang, J. & Fang, F. Removal of SiC at atomic and close-to-atomic scale by nanosecond ultraviolet laser. *Opt. Laser Technol.* **158**, 108863 (2023).

[37] Karnam, D. et al. Plasma mediated ns-laser erosion of SiC monitored using Raman spectroscopy and in-operando LIBS. *Surf. Interfaces*. **46**, 104062 (2024).

[38] Gautam, G. D. & Pandey, A. K. Pulsed nd:yag laser beam drilling: A review. *Opt. Laser Technol.* **100**, 183–215 (2018).

[39] Andrini, G. et al. Activation of Telecom emitters in silicon upon ion implantation and Ns pulsed laser annealing. *Commun. Mater.* **5**, 47 (2024).

[40] Bauer, M. et al. Temperature-depending Raman line-shift of silicon carbide. *J. Raman Spectrosc.* **40**, 1867–1874 (2009).

[41] Reitano, R. et al. Excimer laser induced thermal evaporation and ablation of silicon carbide. *Appl. Surf. Sci.* **96–98**, 302–308 (1996).

[42] Choi, I. et al. Laser-induced phase separation of silicon carbide. *Nat. Commun.* **7**, 13562 (2016). 27901015 10.1038/ncomms13562 PMC5141366

[43] Kasper, C. et al. Influence of irradiation on defect spin coherence in silicon carbide. *Phys. Rev. Appl.* **13**, 044054 (2020).

[44] Lohrmann, A. et al. Single-photon emitting diode in silicon carbide. *Nat. Commun.* **6**, 7783 (2015). 26205309 10.1038/ncomms8783

[45] Fuchs, F. et al. Silicon carbide light-emitting diode as a prospective room temperature source for single photons. *Sci. Rep.* **3**, 1637 (2013). 23572127 10.1038/srep01637 PMC3622138

[46] Niethammer, M. et al. Coherent electrical readout of defect spins in silicon carbide by photo-ionization at ambient conditions. *Nat. Commun.* **10**, 5569 (2019). 31804489 10.1038/s41467-019-13545-z PMC6895084

[47] Roccaforte, F. et al. Structural and electrical properties of Ni∕Ti Schottky contacts on silicon carbide upon thermal annealing. *J. Appl. Phys.* **96**, 4313–4318 (2004).

[48] Vigneshwara Raja, P. et al. Thermal annealing studies in epitaxial 4H-SiC Schottky barrier diodes over wide temperature range. *Microelectron. Reliab.* **87**, 213–221 (2018).

